# Neue digitale Assistenzsysteme: Potenziale in der Viszeralmedizin

**DOI:** 10.1007/s00104-024-02220-9

**Published:** 2025-01-13

**Authors:** Dirk Weyhe

**Affiliations:** Pius-Hospital Oldenburg, Universitätsklinik für Viszeralchirurgie, Georgstr. 12, 26121 Oldenburg, Deutschland

Die Zukunft der Medizin wird vor allem durch den demographischen Wandel und die zunehmend hoch entwickelten, individualisierten und interdisziplinären Therapieoptionen bei gleichzeitig größer werdendem Personalmangel definiert. Das WiFOR Institute analysierte im Auftrag von PricewaterhouseCoopers (PWC) Deutschland, dass 2022 im Gesundheitswesen ein Fachkräftemangel von 290.000 unbesetzten Stellen (6,8 %) bestand und dieser bis zum Jahr 2035 auf 1,8 Mio. (35,4 %) anwachsen wird. Daraus werden starke Versorgungsschwierigkeiten resultieren. Die Robert-Bosch-Stiftung publizierte bereits 2021 die Studie zu „Gesundheitszentren für Deutschland: Wie ein Neustart in der Primärversorgung gelingen kann“. Dabei zeigen deren Projektionen, dass im Jahr 2035 in Flächenlandgebieten wie z. B. Nordwestdeutschland mit einem hausärztlichen Versorgungsgrad von < 75 % eine dramatische Unterversorgung in den Landkreisen bestehen wird. Die Bundesärztekammer beschreibt aus dem Gutachten „Resilienz im Gesundheitswesen“ von 2023 des Sachverständigenrates, dass dem steigenden Bedarf an medizinischer Expertise ein zukünftiger Mangel an Ärzten gegenübersteht. Daher soll in diesem Themenheft ein aktueller Stand und Ausblick gegeben werden, welche Potenziale die neuen digitalen Technologien wie Virtual und Augmented Reality (VR bzw. AR) oder künstliche Intelligenz (KI) besitzen, um diese Herausforderungen der nahen Zukunft zu meistern und den zukünftigen Personalmangel zu kompensieren.

*Kluge et. al.* beschreiben die technischen Voraussetzungen, die notwendig sind, um diese Technologien überhaupt flächendeckend in der Gesundheitsversorgung einsetzen zu können, und formulieren die unter Entwicklerinnen und Entwicklern gerade gängige Definition für die verschiedenen Technologien.

Von *Maier et al.* wird eine breite Übersicht zur Neugestaltung der anatomischen Lehre mit VR- und AR-Einsatz beschrieben. Hier stellt sich immer wieder die Frage, inwieweit die digitale Unterstützung die klassischen Lehrmethoden ergänzt oder sogar ablöst.

*Huber et al.* beschreiben die Potenziale von VR in der Vermittlung spezifischer, operationsrelevanter Strukturen durch 3‑D-Modelle vor dem Hintergrund einer zunehmenden Ressourcenknappheit im Gesundheitswesen, die immer weniger Zeit und Geld für chirurgische Lehre und Weiterbildung nach sich zieht. Dazu präsentieren die Autoren die Ergebnisse einer Studie, die mit einer VR-Umgebung durchgeführt wurde, die im Rahmen des Projekts AVATAR (gefördert durch das Bundesministerium für Bildung und Forschung [BMBF]) entwickelt wurde.

Als wichtige Partner der Viszeralmedizin beschreiben aus gastroenterologischer Sicht *Grade et al.* die potenziellen telemedizinischen Einsatzmöglichkeiten der KI-unterstützten endoskopischen Untersuchungen bei gleichzeitiger konsiliarischer Abstimmung in Echtzeit mittels AR und integrierter Avatarfunktion.

VR, AR und MR sind einander ergänzende und nicht konkurrierende digitale Systeme mit großem Zukunftspotenzial

Die eigene Arbeitsgruppe aus Oldenburg verdeutlicht anhand verschiedener Forschungsergebnisse aus dem BMBF-geförderten VIVATOP-Projekt den Benefit, den der kombinierte Einsatz von VR, AR und 3‑D-Druck haben kann. Dies wird unter anderem am Beispiel einer Studie verdeutlicht, in der die entwickelten Technologien erfolgreich intraoperativ eingesetzt wurden.

## Zusammenfassung

In der Aus- und Weiterbildung bestehen unbestritten signifikante Vorteile beim Einsatz dieser neuen Technologien in der studentischen Lehre sowie der chirurgischen Weiterbildung. Auch wenn einige innovative Technologien bereits verbreitet eingesetzt werden, wie z. B. KI zur Diagnoseunterstützung in der Endoskopie, muss für viele Einsatzgebiete der tatsächliche Nutzen in den klinischen Anwendungsszenarien der Viszeralchirurgie noch multizentrisch gezeigt werden.

**Abb. 1:**
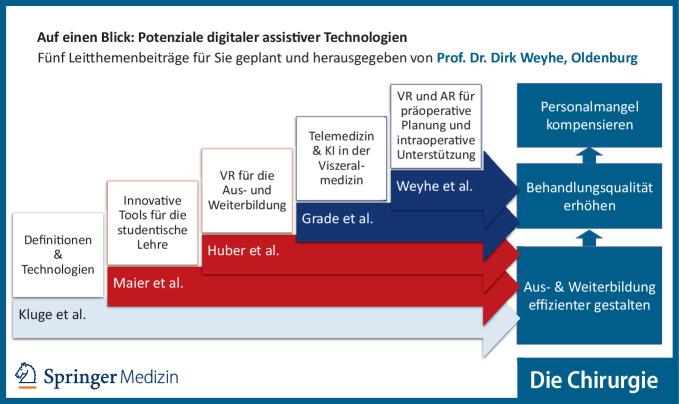
Alle Leitthemenbeiträge auf einen Blick

Insgesamt zeigen die zusammengetragenen Arbeiten auf, wie sich Lehre, Weiterbildung und Behandlung zukünftig verändern werden. Diese Veränderungen führen vor dem Hintergrund des Bedarfs nach zielgerichteter und effektiver Ausbildung und den immer komplexer werdenden Behandlungsoptionen bei gleichzeitig ansteigendem Personalmangel dazu, dass die Beforschung digitaler assistiver Systeme immer wichtiger wird (Abb. 1).

